# The expansion in lymphoid organs of IL-4^+^ BATF^+^ T follicular helper cells is linked to IgG4 class switching in vivo

**DOI:** 10.26508/lsa.201800050

**Published:** 2018-04-05

**Authors:** Takashi Maehara, Hamid Mattoo, Vinay S Mahajan, Samuel JH Murphy, Grace J Yuen, Noriko Ishiguro, Miho Ohta, Masafumi Moriyama, Takako Saeki, Hidetaka Yamamoto, Masaki Yamauchi, Joe Daccache, Tamotsu Kiyoshima, Seiji Nakamura, John H Stone, Shiv Pillai

**Affiliations:** 1 Ragon Institute of MGH, MIT, and Harvard, Massachusetts General Hospital, Harvard Medical School, Boston, MA, USA; 2 Section of Oral and Maxillofacial Oncology, Division of Maxillofacial Diagnostic and Surgical Sciences, Faculty of Dental Science, Kyushu University, Fukuoka, Japan; 3 Department of Internal Medicine, Nagaoka Red Cross Hospital, Nagaoka, Japan; 4 Division of Diagnostic Pathology, Kyushu University Hospital, Fukuoka, Japan; 5 Department of Anatomic Pathology, Kyushu University, Fukuoka, Japan; 6 Laboratory of Oral Pathology, Division of Maxillofacial Diagnostic and Surgical Sciences, Faculty of Dental Science, Kyushu University, Fukuoka, Japan; 7 Division of Rheumatology, Allergy, and Immunology, Massachusetts General Hospital, Harvard Medical School, Boston, MA, USA

## Abstract

Distinct T follicular helper (T_FH_) subsets that influence specific class-switching events are assumed to exist, but the accumulation of isotype-specific T_FH_ subsets in secondary lymphoid organs (SLOs) and tertiary lymphoid organs has not been hitherto demonstrated. IL-4–expressing T_FH_ cells are surprisingly sparse in human SLOs. In contrast, in IgG4-related disease (IgG4-RD), a disorder characterized by polarized Ig class switching, most T_FH_ cells in tertiary and SLOs make IL-4. Human IL-4^+^ T_FH_ cells do not express GATA-3 but express nuclear BATF, and the transcriptomes of IL-4–secreting T_FH_ cells differ from both PD1^hi^ T_FH_ cells that do not secrete IL-4 and IL-4–secreting non-T_FH_ cells. Unlike IgG4-RD, IL-4^+^ T_FH_ cells are rarely found in tertiary lymphoid organs in Sjögren’s syndrome, a disorder in which IgG4 is not elevated. The proportion of CD4^+^IL-4^+^BATF^+^ T cells and CD4^+^IL-4^+^CXCR5^+^ T cells in IgG4-RD tissues correlates tightly with tissue IgG4 plasma cell numbers and plasma IgG4 levels in patients but not with the total plasma levels of other isotypes. These data describe a disease-related T_FH_ subpopulation in human tertiary lymphoid organs and SLOs that is linked to IgG4 class switching.

## Abstract (precis)

T follicular helper cells that secrete IL-4 accumulate in lymphoid organs in IgG4-related disease and are linked to IgG4 class switching.

## Introduction

T follicular helper (T_FH_) cells provide help to B cells during T-dependent immune responses, and they contribute to isotype switching, somatic hypermutation, germinal center (GC) formation, and the selection of high-affinity B cells in the GC (Vinuesa et al, 2005; King et al, 2008; Crotty, 2011). However, how exactly T_FH_ cells provide specificity to class-switching events remains unclear. The idea that unique T_FH_ subsets separately and specifically drive class switching to different Ig isotypes is attractive, but no in vitro or in vivo data exist to firmly establish this notion. Indeed, there have been no studies using multicolor staining approaches to examine human T_FH_ cells in situ in secondary lymphoid organs (SLOs) or tertiary lymphoid organs (TLOs). The possibility that chronic disease states exhibiting polarized isotype switching could provide novel insights into specialized T_FH_ cells served as the rationale for undertaking this study.

Some evidence for specialized T_FH_ subsets, albeit indirect, comes from the studies of circulating human T_FH_ cells that have described three T_FH_ subsets defined on the basis of chemokine receptor expression patterns. The relationship between blood T_FH_-cell subsets and T_FH_ cells in SLOs or TLOs remains unclear. In the studies of Ueno et al (Morita et al, 2011; Ueno et al, 2015) on blood T_FH_ subsets, T_FH1_ cells secrete IFN-γ upon activation and have limited isotype-switching activity when examined in in vitro coculture experiments. T_FH2_ cells secrete IL-4 after many days of in vitro stimulation and can mediate class switching to IgA, IgE, and essentially all IgG isotypes, including IgG4. T_FH17_ cells secrete IL-17 following activation and are equally promiscuous.

Although all T_FH_ cells may have the potential to secrete IL-4, one report has described polarized IL-4–secreting T_FH_ cells in mice in the context of an allergic disease model, and it was suggested that these cells could subsequently differentiate into T_H2_ cells (Ballesteros-Tato et al, 2016). An illuminating study using reporter mice has led to the view that T_FH_ cells initially make IL-21, mature into cells that make IL-21 and IL-4, and eventually make IL-4 alone (Weinstein et al, 2016). These studies also demonstrated that the use of a type 2 response–linked murine pathogen facilitated the induction of IL-4–secreting “T_FH4_” cells. There have been no other reports establishing the existence of functionally distinct T_FH_ subsets in human or murine SLOs or TLOs. Moreover, no cytokine-expressing subset of these cells in tissue sites has been linked so far to any specific disease, nor have T_FH_ subsets been defined that determine specific polarized class-switching events. How the overall transcriptome of an IL-4–secreting T_FH_-cell population may differ from other T_FH_ cell types has also never been determined because such cells have not previously been purified from SLOs or TLOs.

IgG4-related disease (IgG4-RD) is a chronic inflammatory disease characterized by tumescent lesions with characteristic storiform fibrosis, obliterative phlebitis, and a marked lymphoplasmacytic infiltrate that includes a large proportion of IgG4-positive plasma cells (Mahajan et al, 2014; Kamisawa et al, 2015). Circulating expansions of plasmablasts, most of which express IgG4, are a hallmark of active disease (Mattoo et al, 2014). We have shown that circulating plasmablasts are heavily somatically hypermutated, implying that these B-lineage cells are derived from GCs. We have also shown that patients with IgG4-RD exhibit large clonal expansions of CD4^+^ CTLs, the dominant T cells in disease tissues, and that these cells are activated at lesional sites, where they secrete IL-1β, TGF-β1, and IFN-γ (Mattoo et al, 2016; Maehara et al, 2017). Although an increase in blood T_FH2_ cells has been noted in IgG4-RD (Akiyama et al., 2015, 2016), these cells function promiscuously in vitro, as they can facilitate class switching to multiple isotypes in coculture experiments. There is no evidence so far connecting blood T_FH_-cell subsets to any functional counterparts in SLOs or TLOs.

Multicolor staining approaches have hitherto not been used to examine lymphoid organ T_FH_ cells in situ. We show here, using such an approach, that T_FH_ cells making IL-4 are surprisingly sparse in normal human tonsils and mesenteric lymph nodes. However, IL-4– and BATF-expressing T_FH_ cells are dramatically expanded in IgG4-RD TLOs and lymph nodes, frequently making up more than half of all T_FH_ cells. This subset of human T_FH_ cells can be found in association with activation-induced cytidine deaminase (AID)-expressing B cells, is more frequent in extrafollicular sites than in the light zone, and is linked to specific class switching to IgG4.

Isolating T-cell subsets based on their function, such as secretion of their cardinal cytokine, and characterizing their gene expression profiles can help us gain better insights into the biology of specific T_FH_ subsets, as well as define better surface markers for their flow cytometric identification. We therefore used an IL-4 capture assay, followed by FACS sorting, to isolate IL-4–secreting T_FH_ cells from a human tonsil and compared their transcriptomic profiles with CXCR5^hi^PD1^hi^IL-4^−^ tonsillar T_FH_ cells and IL-4–producing CXCR5^−^ non-T_FH_ cells (T_H2_ cells). Our studies validate the notion of functionally distinct T_FH_ subsets, establish a link between tissue and lymphoid organ human IL-4–secreting T_FH_ cells and IgG4-RD, and identify genes that are specifically expressed in and define the human IL-4–secreting T_FH_-cell subset.

## Results

### CD4^+^ICOS^+^IL-4^+^ T cells are sparse in normal tonsils and lymph nodes

**Figure 1.**
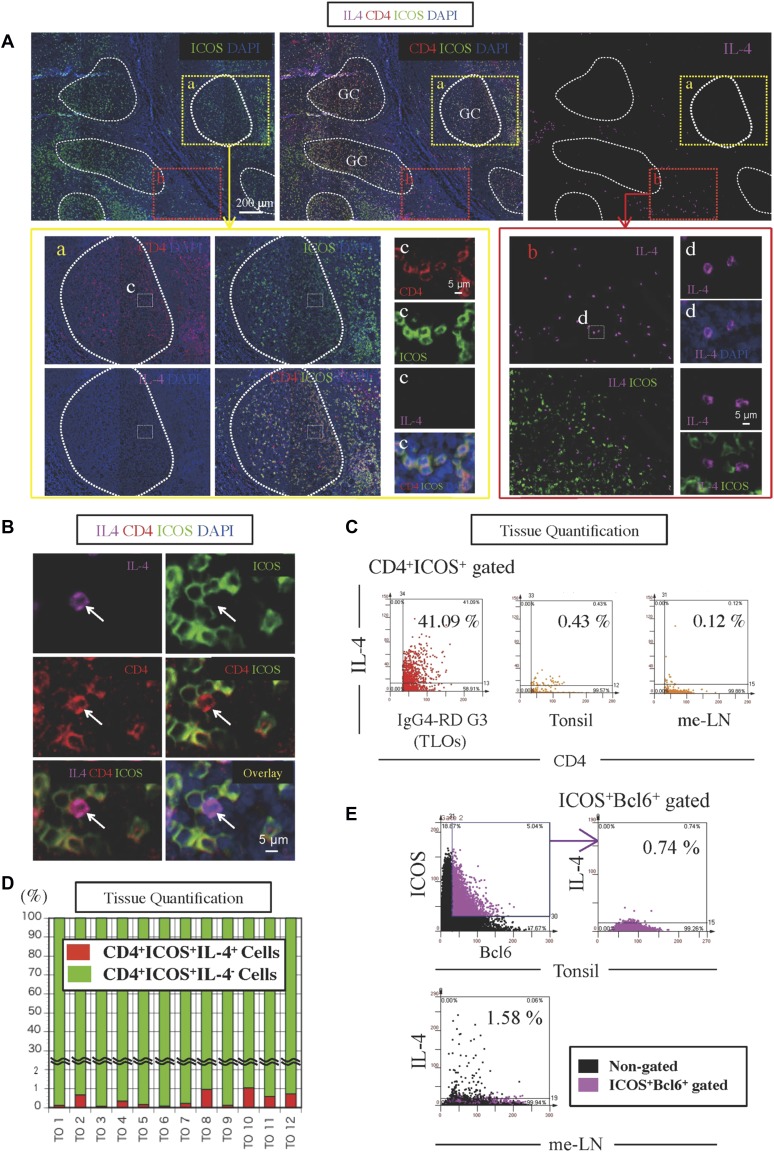
CD4^+^ICOS^+^IL-4^+^ T_FH_ cells are sparse in human SLOs. **(A)** Immunofluorescence staining of CD4 (red), ICOS (green), IL-4 (magenta), and DAPI (blue) in tissues from normal tonsils. The yellow broken line demarcates the area within GCs. The white broken line demarcates the area outside GC. **(B)** Immunofluorescence staining of CD4 (red), ICOS (green), IL-4 (magenta), and DAPI (blue) in tissues from normal tonsils. **(C)** Scatter plots depict the mean fluorescence intensity per cell quantified using TissueQuest software for each fluorescent antibody used to stain tissue from an IgG4-RD patient, normal tonsils, and normal mesenteric lymph nodes. **(D)** CD4^+^ICOS^+^IL-4^+^ (red) and CD4^+^ICOS^+^IL-4^−^ (green) cells were quantified in tissue from 12 tonsils. **(E)** Scatter plots depict the mean fluorescence intensity per cell quantified using TissueQuest software for each fluorescent antibody used to stain normal tonsils and mesenteric lymph nodes.

We initially examined normal tonsils to quantitate CD4^+^ICOS^+^ T cells that express IL-4 in situ. Almost all the IL-4–expressing cells seen in tonsils were CD4^+^ but did not express ICOS (Fig 1A and B). These CD4^+^IL-4^+^ICOS^−^ T cells are presumably T_H2_ cells that reside in T-cell zones in tonsils. Quantitation revealed that CD4^+^ICOS^+^IL-4^+^ T_FH_ cells represent <0.5% of all CD4^+^ICOS^+^ T_FH_ cells in normal tonsils and lymph nodes (Fig 1C). However, quantitative analyses revealed that approximately 40% of CD4^+^ICOS^+^ T_FH_ cells in an IgG4-RD patient expressed IL-4 (Fig 1C). The low frequency of T_FH_ cells that express IL-4 was confirmed in 12 different tonsil specimens (Fig 1D). Relatively low frequencies of IL-4^+^CD4^+^ICOS^+^ and IL-4^+^ICOS^+^Bcl6^+^ T_FH_ cells were also observed in normal mesenteric lymph nodes in addition to tonsillar samples (Fig 1E).

**Figure 2.**
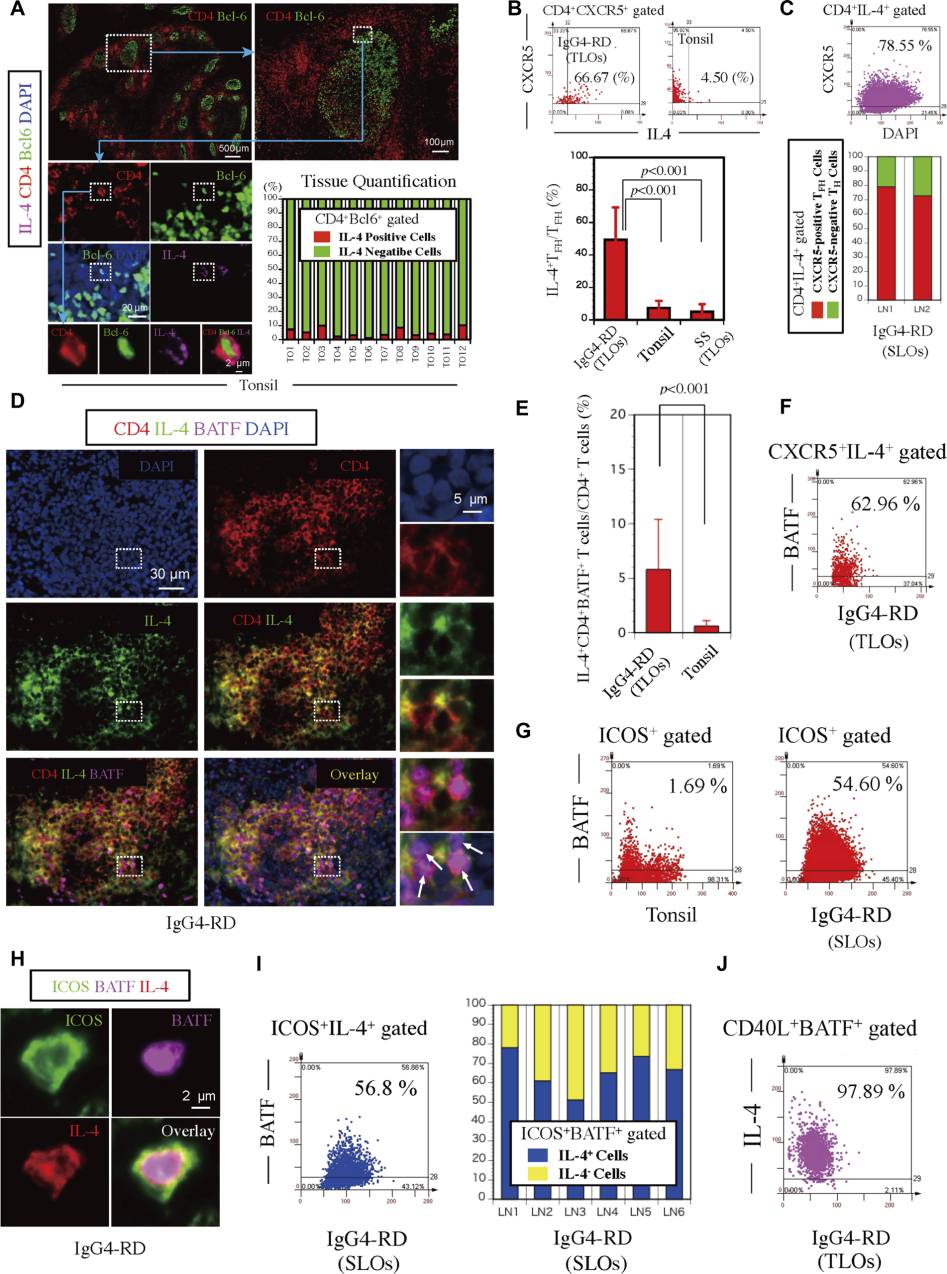
IL-4^+^BATF^+^ T_FH_ cells are rare in normal SLOs but abundant in IgG4-RD. **(A)** Immunofluorescence staining of CD4 (red), Bcl-6 (green), IL-4 (magenta), and DAPI (blue) in tissues from normal tonsils. Quantification of CD4^+^Bcl-6^+^IL-4–positive T_FH_ and CD4^+^Bcl-6^+^IL-4–negative T_FH_ in 12 tonsils. **(B)** Scatter plots depict the mean fluorescence intensity per cell quantified using TissueQuest software for each fluorescent immunostain in IgG4-RD SMGs (G1) and normal tonsils. Quantification of CD4^+^CXCR5^+^IL-4^+^ and CD4^+^CXCR5^+^ T_FH_ cells in 17 IgG4-RD SMGs, 12 tonsils, and 7 SS salivary glands. The *P*-value is based on the Mann–Whitney *U* test. **(C)** Almost all IL-4–expressing CD4^+^ T cells in IgG4-RD lymph nodes were CXCR5^+^ T_FH_ cells. Scatter plots depict the mean fluorescence intensity per cell quantified using TissueQuest software for each fluorescent immunostain in IgG4-RD lymph node (LN1). The quantification of CD4^+^IL-4^+^CXCR5–positive T_FH_ and CD4^+^IL-4^+^CXCR5–negative non-T_FH_ cells in lymph nodes from two patients with IgG4-RD (LN1 and LN2) is shown. **(D)** CD4^+^IL-4^+^BATF^+^ T cells were enriched in IgG4-RD SMGs. Immunofluorescence staining of CD4 (red), IL-4 (green), BATF (magenta), and DAPI (blue) in IgG4-RD SMGs (G12). White arrows indicate CD4^+^IL-4^+^BATF^+^ T cells. **(E)** Quantification of CD4^+^BATF^+^IL-4^+^ T cells and CD4^+^ T cells in 17 IgG4-RD SMGs and 12 normal tonsils. The *P*-value is based on the Mann–Whitney *U* test. **(F)** Scatter plots depict the mean fluorescence intensity per cell quantified using TissueQuest software for each fluorescent antibody used to stain normal tonsils and IgG4-RD lymph node (LN1). **(G)** Scatter plots depict the mean fluorescence intensity per cell quantified using TissueQuest software for each fluorescent immunostain in IgG4-RD SMGs (G3). **(H)** Immunofluorescence staining of ICOS (green), BATF (magenta), and IL-4 (Red) in IgG4-RD LNs (LN1). **(I)** Scatter plots depict the mean fluorescence intensity per cell quantified using TissueQuest software for each fluorescent immunostain in IgG4-RD LNs (LN1). The quantification of ICOS^+^BATF^+^IL-4–positive T and ICOS^+^BATF^+^IL-4–negative T cells in lymph nodes from six patients with IgG4-RD (LN1–LN6). **(J)** Scatter plots depict the mean fluorescence intensity per cell quantified using TissueQuest software for each fluorescent immunostain in IgG4-RD SMGs (G3).

To more directly examine GC T_FH_ cells, we initially localized GCs in 12 different human tonsils using staining for Bcl-6 and then analyzed CD4^+^Bcl-6^+^IL-4^+^ T cells (Fig 2A). These analyses also revealed that CD4^+^Bcl-6^+^IL-4^+^ GC T_FH_ cells represented a very small proportion (ranging from 1% to 10%) of all GC T_FH_ cells.

### CD4^+^CXCR5^+^IL-4^+^ T_FH_ cells are abundant in IgG4-RD but rare in normal SLOs and in Sjögren’s syndrome (SS)

We analyzed SLOs from healthy individuals and affected TLOs in the submandibular glands (SMGs) from IgG4-RD patients with active disease for IL-4–synthesizing T_FH_ cells and focused our analyses in the vicinity of TLO-like structures containing GCs. TLOs with GCs (Ruddle, 2014) were identified using multicolor immunofluorescence approaches (CD4, CD19, Bcl6, and DAPI). In this study, 17 of 25 patients (68%) with IgG4-RD and 7 of 15 patients (47%) with severe SS had TLOs in affected salivary glands (Table S3). GCs within TLOs were both more frequent and larger in the salivary glands from IgG4-RD than severe SS (Table S3). As shown in Fig 2B, CD4^+^CXCR5^+^IL-4^+^ cells were found in human tonsils and lymph nodes but were sparse. In contrast, these CD4^+^CXCR5^+^IL-4^+^ T_FH_ cells were abundant in IgG4-RD. Quantitation of CD4^+^CXCR5^+^IL-4^+^ T_FH_ cells revealed that approximately 67% of CD4^+^CXCR5^+^ T_FH_ cells in an IgG4-RD patient expressed IL-4, whereas fewer than 5% of tonsillar CD4^+^CXCR5^+^ T_FH_ cells expressed IL-4 (Fig 2B). CD4^+^CXCR5^+^IL-4^+^ T_FH_ and CD4^+^ICOS^+^IL-4^+^ T_FH_ cells are clearly far more abundant in IgG4-RD than in normal SLOs in or around TLOs from affected tissues from patients with SS (Fig 2B).

We extended our studies to lymph nodes from patients with IgG4-RD. Quantification revealed that almost all IL-4–expressing CD4^+^ T cells in IgG4-RD lymph nodes were CXCR5^+^ T_FH_ cells (Fig 2C).

### IL-4^+^BATF^+^ T_FH_ cells are rare in normal SLOs but abundant in IgG4-RD

The regulation of IL-4 expression in murine T_FH_ cells is distinct from the mechanisms that drive IL-4 expression in T_H2_ cells. BATF is a transcription factor that may regulate IL-4 secretion by murine T_FH_ cells (Sahoo et al, 2015) but may more broadly help identify T_FH_ cells, some of which do not necessarily express IL-4 (Weinstein et al, 2016). Many CD4^+^IL-4^+^ T cells in the vicinity of GCs in affected IgG4-RD tissues express nuclear BATF (Fig 2D). Not surprisingly, quantification revealed that these IL-4–expressing CD4^+^BATF^+^ T cells are abundant in IgG4-RD tissue lesions but rare in normal SLOs (Fig 2E). Using parallel sections of tissues from an IgG4-RD patient, we noted that CD4^+^BATF^+^IL-4^+^ T cells were located in the same region in which IL-4–expressing CD4^+^CXCR5^+^T_FH_ cells were observed. Quantification of CXCR5^+^BATF^+^IL-4^+^ cells revealed that approximately 60% of CXCR5^+^IL-4^+^ cells in an IgG4-RD patient with TLOs expressed BATF (Fig 2F).

We considered the possibility that the expansion of these IL-4–expressing T_FH_ cells might represent an important disease-related T_FH_ subset and contribute to the specific class-switching event in IgG4-RD. ICOS^+^BATF^+^ T_FH_ cells are far more abundant in IgG4-RD lymph nodes than in normal tonsils (Fig 2G). As shown in Fig 2H, ICOS^+^BATF^+^IL-4^+^ T_FH_ cells were detected in patients with IgG4-RD, and these cells were abundant. Quantification of ICOS^+^BATF^+^IL-4^+^ T_FH_ cells revealed that approximately 60% of ICOS^+^IL-4^+^ T_FH_ cells in an IgG4-RD lymph node patient expressed BATF (Fig 2I). Furthermore, quantification revealed that the majority of ICOS^+^BATF^+^ T_FH_ cells in IgG4-RD lymph nodes expressed IL-4 as well (Fig 2I). Interestingly, quantification of CD40L^+^BATF^+^IL-4^+^ T cells revealed that these cells represented approximately 98% of CD40L^+^BATF^+^ T cells in an IgG4-RD patient (Fig 2J). These data indicate that a large number of lesional IL-4^+^ T_FH_ cells in IgG4-RD express BATF, CD40L, and ICOS.

### IL-4–secreting T_FH_ cells are a distinct population of T_FH_ cells

**Figure 3.**
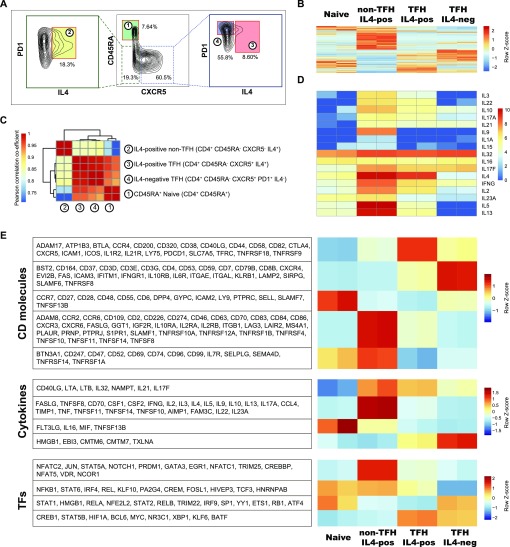
Transcriptomic profiling of IL-4–producing T_FH_ cells from human tonsils. **(A)** Gating strategy used to sort IL-4–secreting and nonsecreting tonsillar CD45RA^+^CXCR5^+^ T_FH_ cells following anti-CD3/anti-CD28 stimulation. CD45RA^+^ CXCR5^−^ cells and CD45RA^−^CXCR5^−^IL-4^+^ cells were sorted as additional controls. **(B)** A heatmap of differentially expressed genes across all four conditions depicting the *Z*-scores for normalized expected read counts. **(C)** A correlation matrix of differentially expressed genes. **(D)** Expression pattern of individual ILs is depicted on a log scale. **(E)** Expression of CD molecules, cytokines, and transcription factors clustered into patterns using *k*-means. *Z*-scores of the expected read counts for each cluster are shown.

Although visualization by multicolor staining permits anatomic localization of T_FH_ cells in tissues, it can provide only limited information about a few expressed proteins in any putative T_FH_-cell subset, and detailed characterization of any specific cytokine-secreting T_FH_ subset is currently lacking. To better understand the biology of IL-4–secreting T_FH_ cells found in lymphoid organs, we performed RNA sequence analysis of viable IL-4–secreting T_FH_ cells from human tonsils. Although the fraction of IL-4–producing T_FH_ cells is low in human tonsils, we were able to purify this subset by starting with 600 million tonsil cells using an IL-4 cytokine capture strategy. To promote cytokine secretion, CD19-depleted lymphocytes from tonsils were stimulated overnight with plate-coated anti-CD3 and anti-CD28 antibodies. We then compared the transcriptomes of FACS-sorted IL-4–producing T_FH_ cells with those of CXCR5^hi^PD1^hi^ T_FH_ cells that did not secrete IL-4, as well as IL-4–secreting CD45RA^−^CXCR5^−^ non-T_FH_ cells obtained from the same tonsil (Fig 3A). CD4^+^ CD45RA^+^ naive cells were also included as an additional control. Of the 26,002 mapped transcripts, 7,792 were differentially expressed across the four conditions (Fig 3B). The IL-4–producing and nonproducing T_FH_ cells were most similar and markedly dissimilar from IL-4–secreting non-T_FH_ cells or naive CD4^+^ T cells (Fig 3C). In contrast to the IL-4–producing non-T_FH_ cells that express high levels of *IL-4*, *IL-5*, and IL-13, reflecting a T_H2_ signature, IL-4–producing T_FH_ cells express only IL-4 but much lower levels of IL-5 and *IL-13* (Fig 3D). To examine lineage-defining genetic regulators and functionally relevant effector molecules, we focused our analysis on differentially expressed CD molecules, transcription factors, and cytokines (Fig 3E). The transcript level of genes critical to T_FH_ function such as *CD40L*, *ICOS*, *CXCR5*, *IL-21R*, and *PD1* was highest in the IL-4–secreting T_FH_ cells. In addition, they also expressed high levels of *CCR4*, *CD200*, *CTLA4*, and *GITR* and low levels of *CD6*, *CD27*, *CD28*, *SELL*, *IL-7R*, and *CD74*. Although the expression levels of these cell surface markers are derived from in vitro activation during cytokine capture, the CD markers specific for cells with an IL-4–secreting T_FH_ phenotype may aid in the specific flow cytometric identification of ex vivo human IL-4–secreting T_FH_ cells in future studies. As expected, CD4^+^ CD45RA^+^ cells had the highest level of *CCR7*, *CD27*, and *CD62L*. The high expression levels of multiple chemokines, cytokines, and their receptors including *CCR2*, *CCR6*, *CXCR3*, *CXCR6*, *IL-2RA*, *IL-2RB*, *IL-10RA*, *CCL4*, *IFNG*, *IL-2*, *IL-3*, *IL-4*, *IL-5*, *IL-9*, *IL-10*, *IL-17A*, *IL-22*, and *IL-23A* within the IL-4–producing CD4^+^CD45RA^−^ non-T_FH_ cells perhaps indicate the heterogeneity among these cells and may reflect the overlap and plasticity between T_H1_, T_H2_, and T_H17_ subsets that are seen following TCR stimulation in the absence of polarizing cytokines (i.e., anti-CD3 and anti-CD28 alone). Interestingly, the IL-4–producing T_FH_ cells express the highest levels of *BCL6* and *BATF* in this comparison but not transcription factors related to T_H2_ differentiation such as *GATA3*, *STAT5A*, and *PRDM1 (BLIMP1)*, which are instead abundantly expressed in the IL-4–producing non-T_FH_ population, which is likely enriched for T_H2_ cells. Furthermore, the expressions of all the markers that we studied using immunofluorescence in IL-4–expressing T_FH_ cells from IgG4-RD tissues (BCL6, ICOS, IL-4, CXCR5, BATF, GATA3, and PD1) were consistent with the RNA sequence observations on the tonsillar IL-4–secreting T_FH_ cells.

### CD4^+^CXCR5^+^IL-4^+^ T_FH_ cells are mainly outside GCs in IgG4-RD and sometimes physically associate with AID-expressing B cells

**Figure 4.**
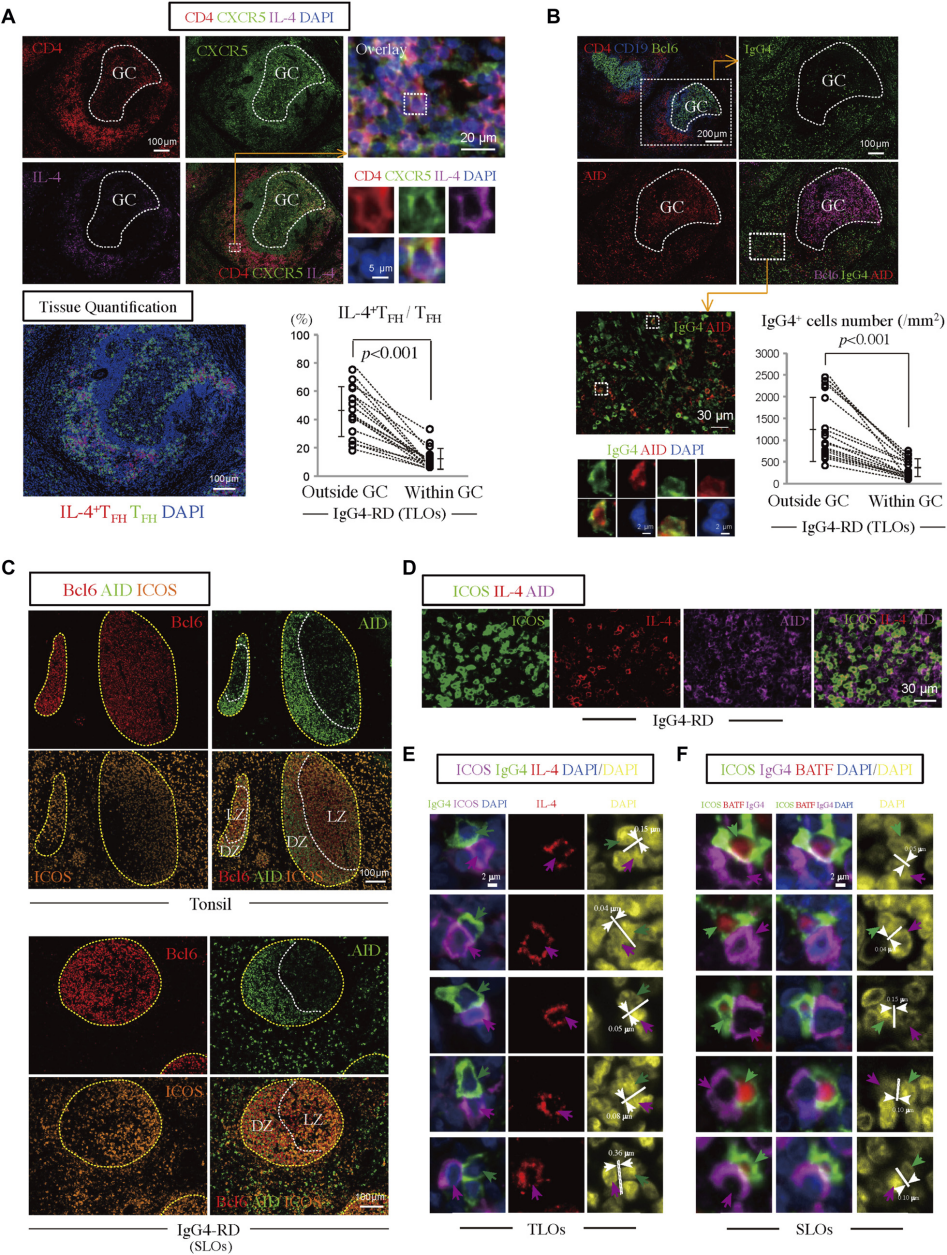
CD4^+^CXCR5^+^IL-4^+^ T_FH_ cells are abundant around GCs in IgG4-RD and sometimes physically contact AID-expressing B cells. **(A)** CD4^+^CXCR5^+^IL-4^+^ T cells were enriched around TLOs in IgG4-RD SMGs. Immunofluorescence staining of CD4 (red), IL-4 (magenta), CXCR5 (green), and DAPI (blue) in IgG4-RD SMGs (patient G3). Quantification of CD4^+^CXCR5^+^IL-4^+^ T_FH_ cells and CD4^+^CXCR5^+^ T_FH_ cells comparing those outside and within GCs from each of five different areas in TLOs of 17 patients with IgG4-RD (G1–G17). The *P*-value is based on the Mann–Whitney *U* test. **(B)** IgG4^+^B cells were enriched outside GC, especially some these cells express AID. Immunofluorescence staining of CD4 (red), CD19 (blue), and Bcl6 (green) in IgG4-RD SMGs (patient G3). Immunofluorescence staining of AID (red), Bcl6 (magenta), IgG4 (green), and DAPI (blue) in IgG4-RD SMGs (patient G3). The numbers of IgG4^+^ cells (per square micrometer) outside and within GCs were quantified from each of five different areas in TLOs of 17 patients with IgG4-RD (G1–G17). The *P*-value is based on the Mann–Whitney *U* test. **(C)** AID-expressing B cells outside GCs in IgG4-RD lymph nodes were abundant. Immunofluorescence staining of Bcl6 (red), AID (green), and ICOS (orange) in a normal tonsil and IgG4-RD lymph node. **(D)** Immunofluorescence staining of ICOS (green), IL-4 (red), and AID (magenta) in IgG4-RD SMGs. **(E)** Immunofluorescence staining of IgG4 (green), ICOS (magenta), IL-4 (red), and DAPI (blue) in TLOs with an IgG4-RD patient (G3). Magenta arrows indicate ICOS^+^IL-4^+^ T_FH_ cells. Green arrows indicate IgG4^+^ B cells. A number of IL-4–expressing T_FH_ cells and IgG4+ B cells formed close and extensive intercellular plasma membrane contacts. The distance was measured between the edge of each IgG4^+^ B cell nucleus to the edge of the closest IL-4–expressing T_FH_-cell nucleus, and an internuclear distance of less than 0.4 μm was indicative of a T-B conjugate. **(F)** Immunofluorescence staining of ICOS (green), IgG4 (magenta), BATF (red), and DAPI (blue) in the lymph nodes of an IgG4-RD patient. Green arrows indicate ICOS^+^BATF^+^ T_FH_ cells. Magenta arrows indicate IgG4^+^ B cells. A number of BATF^+^ICOS^+^ T_FH_ cells and IgG4^+^ B cells formed close and extensive intercellular plasma membrane contacts. The distance was measured between the edge of each IgG4^+^ B-cell nucleus to the edge of the closest BATF^+^ICOS^+^ T_FH_-cell nucleus. All internuclear distances in this figure were <0.2 μm. LZ, light zone; DZ, dark zone.

As shown in Fig 4A, CD4^+^CXCR5^+^IL-4^+^ T_FH_ cells were abundant in affected IgG4-RD tissues and were mainly outside GCs but were also located in the light zone within GCs. Using parallel tissue sections, we noted that IgG4-positive B cells were abundant outside GCs in the same region in which IL-4–secreting T_FH_ cells were observed. We quantitated IL-4–expressing T_FH_ cells and IgG4-expressing B cells within and outside GCs and observed that the majority of both cell types reside outside GCs (Fig 4A and B).

It is generally accepted that CD40L-CD40 signaling induces AID and that specific cytokines target selected switch regions. Do the IL-4–expressing T_FH_ cells and AID-expressing B cells make physical contact with one another? We examined lymph nodes and TLOs from IgG4-RD patients to determine whether ICOS^+^ T_FH_ cells are in physical contact with AID-expressing B cells or IgG4-expressing B cells in situ. AID-expressing B cells could be visualized outside GCs in IgG4-RD sections (Fig 4B and C); we noted that IgG4-expressing B cells outside GCs often express AID (Fig 4B). We also noted the existence of IL-4^+^ICOS^+^ T_FH_ cells in cell–cell contact with AID-expressing B cells (Fig 4D). As has been reported earlier, much of the AID staining seen is cytosolic (Cattoretti et al, 2006). Furthermore, we noted the existence of IL-4^+^ICOS^+^ and BATF^+^ICOS^+^ T_FH_ cells that are in cell–cell contact with IgG4-expressing B cells (Fig 4E and F), confirming visual contact with nuclear distance measurements. AID^+^IgG4^+^ B cells were also visualized within and outside GCs in TLOs in IgG4-RD.

### CD4^+^CXCR5^+^IL-4^+^ T_FH_ cells and CD4^+^IL-4^+^BATF^+^ T cells are enriched in IgG4-RD, and their proportions are tightly linked to serum IgG4 levels and the proportion of IgG4-positive plasma cells in tissues

**Figure 5.**
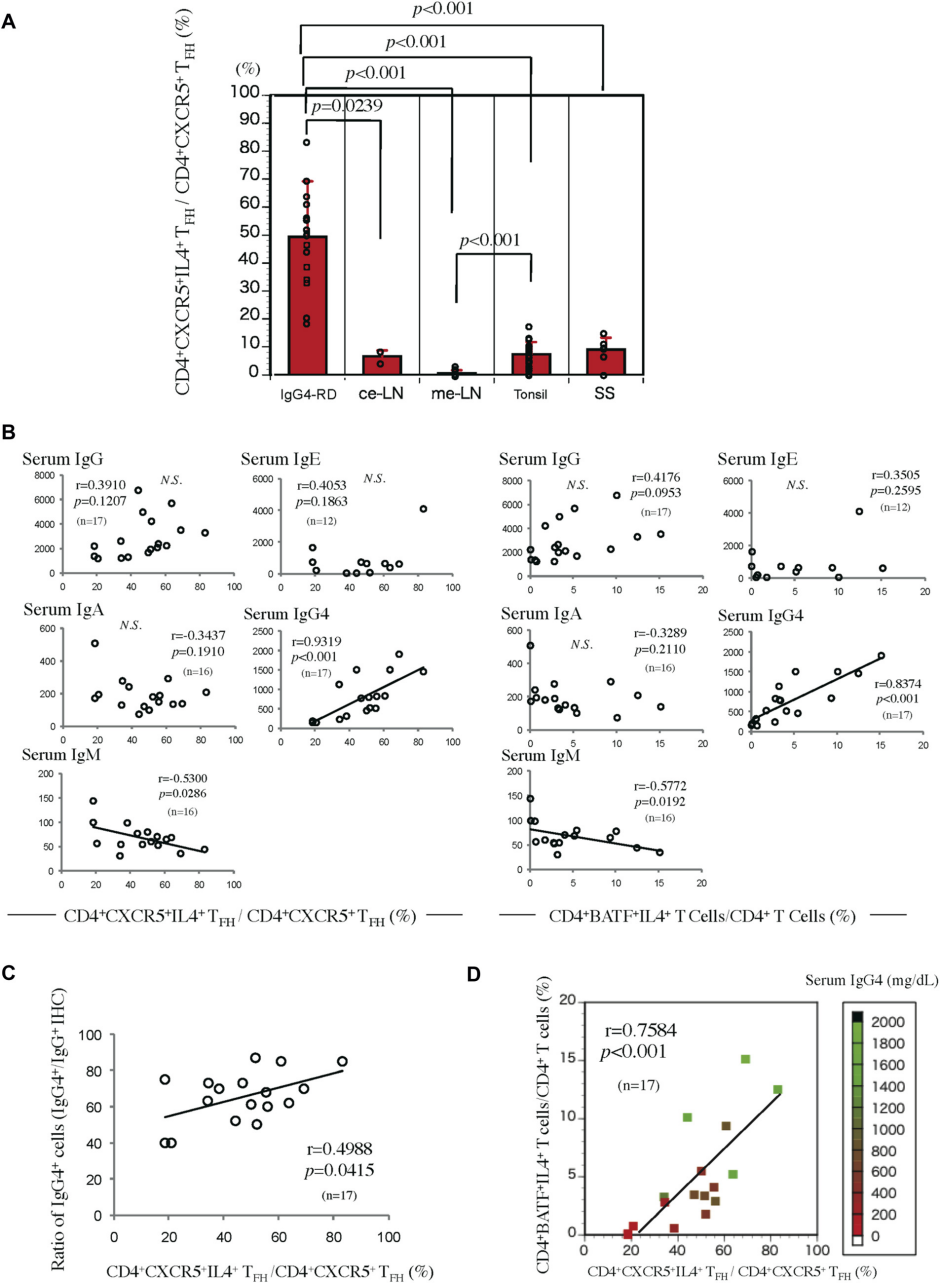
CD4^+^CXCR5^+^IL-4^+^ T_FH_ cells and CD4^+^IL-4^+^BATF^+^ T cells are enriched in IgG4-RD, and their proportions are tightly linked to serum IgG4 levels and IgG4-positive plasma cells. **(A)** Quantification of CD4^+^CXCR5^+^IL-4^+^ and CD4^+^CXCR5^+^ T_FH_ cells in 17 IgG4- RD SMGs, seven SS LSGs, 12 tonsils, two cervical lymph nodes, and three mesenteric lymph nodes. The *P*-value is based on the Mann–Whitney *U* test. **(B)** Correlations of the proportion of CD4^+^CXCR5^+^IL-4^+^ T_FH_ cells and CD4^+^BATF^+^IL-4^+^ T cells in SMGs from patients with IgG4-RD and their serum IgG, IgA, IgE, IgM, or IgG4 levels. The *r* and *P*-value were determined using Spearman’s rank correlations. **(C)** Correlations of the proportions of CD4^+^CXCR5^+^IL-4^+^ T_FH_ cells in SMGs from patients with IgG4-RD and their ratio of IgG4^+^/IgG^+^ cells (n = 17). The *r* and *P*-value were determined using Spearman’s rank correlations. **(D)** The frequency of CD4^+^CXCR5^+^IL-4^+^ T_FH_ cells in SMGs from patients with IgG4-RD (n = 17) correlated with the frequency of CD4^+^BATF^+^IL-4^+^ T cells and serum IgG4 concentrations. The *r* and *P*-value were determined using Spearman’s rank correlations.

As shown in Fig 5A, only a small proportion of CD4^+^CXCR5^+^ T_FH_ cells in tonsils, mesenteric and cervical lymph nodes from normal individuals, and also in or around TLOs from affected tissues from patients with SS synthesize IL-4. These data argue that IL-4 expression is not generally a part of the T_FH_ phenotype in cells located in SLOs or TLOs. Instead, what is more likely is that a subset of activated T_FH_ cells that express IL-4 expands considerably in a disease in which there is prominent IgG4 class switching, but these cells are not abundant around GCs in healthy people or in TLOs from other diseases in which there is no prominent class switching to the IgG4 or IgE isotypes. The proportion of T_FH_ cells that express IL-4 in disease tissue correlates very strongly with the serum IgG4 levels in IgG4-RD patients but not with total serum IgG, IgE, or IgA levels (Fig 5B). A negative correlation with serum IgM levels was also observed (Fig 5B). The proportion of CD4^+^CXCR5^+^ cells that express IL-4 in IgG4-RD also correlates with the proportion of IgG4-expressing plasma cells in disease tissues (Fig 5C). The proportions of CD4^+^BATF^+^IL-4^+^ T cells correlate with CD4^+^CXCR5^+^IL-4^+^ T_FH_ cells and with serum IgG4 levels (Fig 5D).

## Discussion

In T-dependent immune responses, it is widely accepted that specific cytokines induce the transcription of selected switch regions so that distinct Ig gene loci are targeted by AID for the induction of cytidine deamination and double-strand break formation. However, a link between subsets of T_FH_ cells and specific isotype switching events has not been established.

We found that very few T_FH_ cells in healthy tonsils or lymph nodes synthesize IL-4. These findings are broadly consistent with published data on human tonsillar T_FH_ cells using different approaches (Ma et al, 2009; Bentebibel et al, 2011; Kroenke et al, 2012). Bentebibel et al (2011) reported that T_FH_ cells from outside and within GCs secrete IL-4 when cocultured in vitro with B cells. However, as these data did not provide information at the single-cell level, the percentage of T_FH_ cells capable of secreting IL-4 could not be surmised from this report. Kroenke et al used flow cytometry to quantitate intracellular cytokines in in vitro restimulated T_FH_ cells from tonsils. Following restimulation with PMA and ionomycin, <3% of tonsillar T_FH_ cells expressed IL-4 (Kroenke et al, 2012). Ma et al (2009) also used flow cytometry to measure cytokine expression by tonsillar T_FH_ cells following PMA and ionomycin exposure and observed that up to 16% of CXCR5^hi^ T_FH_ cells expressed IL-4. Our in situ data, based on direct examination of IL-4 in T_FH_ cells, rather than on in vitro restimulation, indicate that only a small fraction of T_FH_ cells in normal human SLOs produce IL-4. These data suggest that IL-4–secreting T_FH_ cells are relatively rare in human tonsils to begin with, and only a small fraction of healthy tonsillar T_FH_ cells have presumably been restimulated in vivo to synthesize IL-4, as evidenced by our in situ studies. In IgG4-RD, in contrast, a very large proportion of T_FH_ cells that can secrete IL-4 infiltrate tissues and SLOs, and because these cells have likely been restimulated in vivo, they can be seen to express IL-4 in our in situ analyses.

Human T_FH_ subsets have only been described in the context of putative circulating memory CD4^+^ T cells and not in a tissue context. Studies on T_FH_ cells in disease have generally focused on an analysis of the blood. We examined human T_FH_ cells in situ using multicolor quantitative immunofluorescence approaches, focusing on lymph nodes and TLOs in tissues of patients with a disease in which there is a pronounced switch to the IgG4 isotype. The proportions of CD4^+^CXCR5^+^IL-4^+^ T cells, and their likely equivalent, CD4^+^BATF^+^IL-4^+^ T cells, are markedly increased in IgG4-RD (with most IL-4–expressing T_FH_ cells located in lymphoid cuffs just outside GCs), and these proportions correlate well with serum IgG4 levels and the proportion of IgG4-positive cells in disease tissues. Our data link IL-4–expressing T_FH_ cells to IgG4 class switching and also indirectly argue that most class switching likely occurs outside GCs.

IL-4–secreting T_FH_ cells have never been purified from lymphoid organs and cannot be distinguished in human lymphoid organs by the expression of specific chemokine receptors. To reliably characterize a specific cytokine-secreting T_FH_ population, we used a specific IL-4 capture assay and compared the transcriptomes of IL-4–producing human tonsillar T_FH_ cells with those of non-T_FH_ cells from the tonsil that secrete IL-4 and human tonsillar PD-1^hi^ T_FH_ cells that do not secrete IL-4. IL-4–secreting T_FH_ cells stand out as a distinct subset. It is reasonable to consider that these IL-4–secreting BATF-expressing T_FH_ cells, which do not express GATA-3, represent an activated T_FH_ population or subset in SLOs and TLOs that are expanded in disease that may contribute to class switching to IgG4. Unlike T_H2_ cells, which produce IL-4, IL-5, and IL-13, the IL-4–producing tonsillar T_FH_ cells produce IL-4 and IL-10 but not IL-5 or IL-13. This subset of T_FH_ cells has a unique transcriptional profile and a distinct set of surface markers that will facilitate future functional studies.

The mechanisms and signals by which antibodies class switch to IgG4 are poorly understood. CD4^+^ T cells can activate IgM-positive B cells to switch to IgG4 and IgE in the presence of added IL-4 (Gascan et al, 1991). It has been argued based on in vitro studies that IL-10 may contribute to IgG4 class switching indirectly by somehow facilitating IL-4–mediated switching to IgG4 in preference to IgE (Jeannin et al, 1998). No molecular or cellular explanation has emerged for this in vitro phenomenon. Arguments have also been made that switching to IgG4 is a result of repeated division, and that switching progresses sequentially along chromosome 14 from IgG1 to IgG3 to IgG2 to IgG4 (Tangye et al, 2002; Jackson et al, 2014). We do not presume that IL-4–secreting T_FH_ cells alone contribute to IgG4 class switching, although it is possible that the same subset of IL-4–secreting T_FH_ cells that we have characterized might, in certain contexts, make enough IL-10 to facilitate this switching event. Perhaps more efficient purification of this T_FH_ subset using surface markers inferred from our transcriptomic data will facilitate future functional studies on IL-4–secreting T_FH_ cells. Tissue sources of IL-10 or of other factors that work in concert with IL-4 from these specific T_FH_ cells will also need to be explored in future studies.

These studies are the first demonstration of an in situ expansion of what may be construed to be a human T_FH_ subset. Many diseases exist in which IgE dominates in the absence of IgG4 and vice versa. Our data indicate that only a fraction of T_FH_ cells in SLOs and TLOs, mainly outside GCs, express BATF and IL-4. However, we have shown here that these IL-4–expressing T_FH_ cells accumulate in TLOs of patients with IgG4-RD. There is therefore a disease-specific increase in the accumulation of a subset of T_FH_ cells and a tight association between the numbers of these cells and class switching, specifically to IgG4. Whether there are further subsets of BATF- and IL-4–expressing T_FH_ cells that separately facilitate IgG4 or IgE class switching remains to be ascertained using single-cell approaches.

## Materials and Methods

### Study population

SMGs were obtained from 17 Japanese patients with IgG4-RD, from affected lymph nodes of 6 patients with IgG4-RD, and from lacrimal and salivary glands (LSGs) of 7 patients with active SS. In addition, 2 unaffected cervical lymph nodes, 3 mesenteric lymph nodes, and 12 healthy human tonsils were obtained, which were histologically normal. All these patients with IgG4-RD had been followed up between 2007 and 2015 at the Department of Oral and Maxillofacial Surgery of Kyushu University Hospital, a tertiary care center. Open SMG biopsies were obtained from patients with IgG4-RD (Moriyama et al, 2014). IgG4-RD was diagnosed according to the following criteria (Umehara et al, 2012): (i) persistent (longer than 3 mo) symmetrical swelling of more than two lacrimal and major salivary glands; (ii) high (>135 mg/dl) serum concentrations of IgG4; and (iii) infiltration of IgG4-positive plasma cells into tissue (IgG4^+^ cells/IgG^+^ cells >40%), as determined by immunostaining. All SMGs from patients with IgG4-RD had histopathologic features of IgG4-RD. Age, sex, serum Ig, and specific autoantibody levels of 17 patients with IgG4-RD (G1–G17) whose affected salivary gland biopsies were analyzed by ex vivo in situ immunofluorescence studies are summarized in Table S1A. Age, sex, serum Ig, and specific autoantibody levels of six patients with IgG4-RD (LN1–LN6) whose affected lymph node biopsies were analyzed by ex vivo in situ immunofluorescence studies are summarized in Table S2.

Table S1 Background information and clinical and serological findings for 17 patients with IgG4-RD and seven patients with SS whose affected salivary gland biopsies were analyzed in ex vivo in situ immunofluorescence studies.

Table S2 Background information and serological findings for six patients with IgG4-RD whose affected lymph node biopsies were analyzed by ex vivo in situ immunofluorescence studies.

Each patient with SS exhibited objective evidence of salivary gland involvement based on the presence of subjective xerostomia and a decreased salivary flow rate, abnormal findings on parotid sialography, and focal lymphocytic infiltrates in the LSGs by histology (Vitali et al, 2002). All patients with SS were severe cases and had developed abundant TLOs in each salivary gland. None of the patients with IgG4-RD and SS had a history of treatment with steroids or other immunosuppressants, infection with HIV, hepatitis B virus, hepatitis C virus, or sarcoidosis, and none had evidence of malignant lymphoma at the time of the study. Age, sex, serum Ig, and specific autoantibody levels of patients with SS whose salivary gland biopsies were analyzed by in situ immunofluorescence are summarized in Table S1B.

Normal human mesenteric lymph node and tonsil patients were obtained from Massachusetts General Hospital. Normal human cervical lymph nodes were obtained from the Department of Oral and Maxillofacial Surgery of Kyushu University Hospital.

The study protocol was approved by the Ethics Committee of Kyushu University, Japan, and the Institutional Review Board at Massachusetts General Hospital. All patients provided written informed consent before participating in the study.

### Multicolor immunofluorescence staining

Tissue samples were fixed in formalin, embedded in paraffin, and sectioned. These specimens were incubated with the following antibodies: anti-AID (clone: ZA001; Invitrogen), anti-IgG4 (clone: ab109493; Abcam), anti-ICOS (clone: 89601; Cell Signaling Technology), anti-IL-4 (clone: MAB304; R&D Systems), GATA3 (clone: CM405A; Biocare), CXCR5 (clone: MAB190; R&D Systems), Bcl-6 (clone: CM410A,C; Biocare), BATF (clone: 10538; Cell Signaling Technology), CD4 (clone: CM153A; Biocare), and CD19 (clone: CM310 A,B; Biocare), followed by incubation with secondary antibodies using a SuperPicTure Polymer Detection Kit (Invitrogen) and an Opal 3-Plex Kit (Fluorescein, Cyanine3, and Cyanine5). The samples were mounted with ProLong Gold Antifade mountant containing DAPI (Invitrogen).

### Microscopy and quantitative image analysis

Images of the salivary gland specimens were acquired using the TissueFAXS platform (TissueGnostics). For quantitative analysis, the entire area of the tissue involved by the lymphoplasmacytic infiltrate was acquired as digital grayscale images in four channels with filter settings for FITC, Cy3, and Cy5 in addition to DAPI. Cells of a given phenotype were identified and quantitated using TissueQuest software (TissueGnostics), with cutoff values determined relative to the positive controls. This microscopy-based multicolor tissue cytometry software permits multicolor analysis of single cells within tissue sections similar to flow cytometry. The principle of the method and the algorithms used have been described in detail elsewhere (Ecker & Steiner, 2004).

### Evaluation of TLOs with IgG4-RD and SS

TLOs with GCs (Ruddle, 2014) were identified using multicolor immunofluorescence approaches (CD4, CD19, Bcl6, and DAPI). In this study, SMG and LSG tissue sections from 25 patients with IgG4-RD and 15 patients with severe SS were evaluated. Distinct Bcl-6^+^ GCs were observed in affected IgG4-RD tissues. Bcl-6^+^ GC B cells in TLOs were within B-cell follicles, delineated using antibodies to CD19. 17 of 25 patients (68%) with IgG4-RD had TLOs in affected salivary glands. Age, sex, serum Ig, and specific autoantibody levels of 17 patients with IgG4-RD (G1–G17) whose affected salivary gland biopsies were analyzed by in situ immunofluorescence are summarized in Table S1A. 7 of 15 patients (47%) with severe SS had TLOs in affected salivary glands. Age, sex, serum Ig, and specific autoantibody levels of patients with SS whose salivary gland biopsies were analyzed in this study are summarized in Table S1B.

The number of TLOs with GCs and the size of GCs in each TLO were evaluated in 4 mm^2^ sections from five different areas of 17 patients with IgG4-RD (G1–G17) and 7 patients with SS (SS1–SS7) (Table S3).

Table S3 Difference in TLOs between patients with IgG4-RD and SS. †Significance of the difference between the two groups was determined using χ^2^ tests. ††Significance of the difference between the two groups was determined using *t* tests.

### IL-4 capture assay and cell sorting

600 million cells from human tonsils were resuspended in Miltenyi magnetic-activated cell sorting buffer and stained with biotinylated anti-human CD19 (clone: HIB19; BioLegend) on ice for 25 min. Cells were washed once in magnetic-activated cell sorting buffer and incubated with anti-biotin microbeads (Miltenyi Biotec) for 25 min on ice. Cells were then loaded on two separate Miltenyi LS columns (at 300 million cells per column), and the flow-through was collected as the B cell–depleted fraction. These cells were spun down and stimulated overnight with plate-coated anti-CD3 (5 μg/ml) and anti-CD28 (5 µg/ml) antibodies. The stimulated cells were then enriched for IL-4–secreting cells using the vendor’s protocol (IL-4 Secretion Assay Kit; Miltenyi Biotec, #130-054-101). The enriched IL-4^+^ cell fraction was surface stained with antibodies against CD4 (BioLegend, #317420), CD45RA (clone: H100; BioLegend, #304122), CXCR5 (clone: J252D4; BioLegend, #356920), and PD1 (clone: EH12.2H7; BioLegend, #329924). The following populations were viably sorted using FACSAria2 (Becton Dickinson) directly into Qiagen RLT-plus buffer (with 1% 2-ME): (i) IL-4–secreting CD4^+^CXCR5^+^ T_FH_ cells, (ii) IL-4–secreting CD4^+^CD45RA-CXCR5^−^ T_H2_ cells, (iii) IL-4–negative CD4^+^CXCR5^+^PD1^+^ T_FH_ cells, and (iv) IL-4–negative CD45RA^+^ cells.

### Trancriptomic analyses

Total RNA was isolated from the FACS-sorted cells using the RNeasy plus Micro Kit (Qiagen). RNA-sequencing libraries were prepared as previously described (Picelli et al, 2013). Briefly, whole transcriptome amplification and tagmentation-based library preparation were performed using the SMART-Seq2 protocol, followed by 35-bp paired-end sequencing on a NextSeq 500 instrument (Illumina). 5–10 million reads were obtained from each sample and aligned to the University of California, Santa Cruz hg38 transcriptome. Gene expression was calculated using RSEM as previously described (Li & Dewey, 2011). The EBSeq package was used to identify differentially expressed genes with a posterior probability of differential expression >0.95 (Leng et al, 2013). Our analysis focused on cytokines, transcription factors, and CD molecules. Cytokines and transcription factors were obtained using the gene ontology terms “GO:0003700” and “GO:0005125,” respectively. Transcription factors pertaining to immune cells were filtered using a literature-based gene prioritization approach with the query terms “immune response,” “immunity,” “T cells,” and “lymphocytes” (Fontaine et al, 2011). The data discussed in this publication have been deposited in National Center for Biotechnology Information’s Gene Expression Omnibus and are accessible through GEO Series accession number GSE111968.

### Statistical analyses

Differences between groups were determined using χ^2^ tests, *t* tests, Mann–Whitney *U* tests, and Spearman’s rank correlations. All statistical analyses were performed using JMP Pro software, version 11 (SAS Institute) for Mac. *P*-values < 0.05 were considered statistically significant. Nonsignificant differences were not specified. In all figures, bar charts and error bars represent means ± SEM.

## Supplementary Information

Supplementary Information is available at https://doi.org/10.26508/lsa.201800050.

## Supplementary Material

Reviewer comments
